# Type 1 diabetes research: Newer approaches and exciting developments

**DOI:** 10.4103/0973-3930.53119

**Published:** 2009

**Authors:** C. B. Sanjeevi

**Affiliations:** Karolinska Institutet, Diabetes and Endocrinology Division, Molecular Immunogenetics Group at Center for Molecular Medicine; Department of Molecular Medicine and Surgery, Karolinska Hospital, Building L5:01, S-17176 Stockholm, Sweden

Dr. O. Raha and others[1] have written an extensive and exhaustive report on type 1 diabetes (T1D) research. They have addressed the role of genetics, environment, bio-markers as well as prevention.

In this Editorial I would like to bring to the attention of readers three important aspects of T1D research in the area of T1D incidence, role of viruses and some new approaches taken by the Type 1 Diabetes Genetics Consortium (T1DGC). I would further like to discuss current approaches in beta cell preservation and regeneration of new beta cells that are likely to benefit not only newly diagnosed T1D patients but also those who developed T1D in the past.

A recent report published in Lancet[2] indicates that T1D incidence has doubled and is likely to double from now to year 2020 in those aged below five years and increase 70% in those below 15 years. There is an annual increase in incidence in each of the countries studied in Europe and a trend towards an increase among younger children. Interestingly, lower incidence countries in Eastern Europe have seen a rapid increase in yearly incidence (eg: Romania and Poland). This increase in incidence cannot be attributed to the changing genetic makeup of the population. What is changing is the lifestyle and practice of better hygiene. Environment and lifestyle seem to be changing and this is most likely the cause for increase. The reasons attributed for the increase in European population, based on the analytical epidemiological studies, are (i) modern lifestyle habits (ii) increased height and weight development (iii) increased caesarean section deliveries or (iv) reduced frequency of early infections. The Eastern Europe is actually playing catch-up with Western Europe and the increased incidence also correlates with increased Gross Domestic Product (GDP) of those countries. This becomes highly relevant for Indian scenario as India is in a similar situation.

The National Institute of Health (NIH) is pioneering some of the world-wide efforts in T1D genetic studies under the ‘T1DGC’. More information on this can be found on the website: www.t1dgc.org where one can find the latest in the Genome Wide Association Studies (GWAS) from T1D samples collected from all over the world. This effort has resulted in the identification of 40 new loci[3] for T1D. This is in addition to the four known non-HLA loci (INS, CTLA4, PTPN22 and IL-2RA). Off all the loci, association of HLA stands out as the only locus with highest lod scores whereas the rest come close to 1.5 to two. The DNA or data from the T1DGC is available for researchers worldwide to perform more studies and more data analysis to come up with newer findings. A new database for T1D research has been setup in this initiative called ‘T1Dbase’ where one can find a lot of information and cross references (www.t1dbase.org).

Viruses are major players under environmental factors implicated in the etiology of the disease. A recent report in the May 28 issue of Science[4] identified four genetic variations in IFIH1 which independently lowered the risk of T1D by more than 50%. A helicase enzyme, IFIH1, triggers the secretion of interferons in response to viral infections, which in turn up-regulate the MHC class I molecules in beta cells (post-viral infection) leading to T cell response and beta cell death. Disabling the expression of IFIH1 lowers the risk for T1D. This is important in the context of earlier findings that suggest that entero-viruses (implicated in the etiology of T1D), which replicate faster are associated with T1D and those which replicate slower are not.

These studies in the etiology of the disease and other NIH funded studies on newborn screening and follow-up (TEDDY consortium or the **T**he **E**nvironmental **D**eterminants of **D**iabetes in the **Y**oung) identify the need for prevention. This study identifies environmental factors pre-disposing to or protective against islet auto-immunity and T1D.[5] Between 2004-2009 TEDDY has been screening 360000 newborns from general population from six centers in the world (three in Europe and three in USA) and families already affected by T1D to identify 17804 children with high risk HLA-DR and DQ genotypes. Of those 7801 will be enrolled in prospective follow-up beginning before the age of 4.5 months. As of May, 2008, TEDDY has screened 25,000 newborns and enrolled 5,000 infants. These infants are screened every three months, up to the age of four years and every six months till they reach 15 years. Blood samples are collected in every visit for detection of candidate infectious agents and nutritional biomarkers. Monthly stool samples are also collected for screening of infectious agents. The primary end points of the study include (a) appearance of one or more autoantibodies confirmed in 2 consecutive visits and (b) development of T1D. More details of the study can be found on the website: http://teddy.epi.usf.edu/

Several vaccine/immune intervention candidates are in Phase III studies and we hope that the intervention options for beta cell preservation and regeneration becomes a reality in few years from now.

These candidates include:

**Antigen based therapy**

- Alum Formulated GAD
- DiaPep277

**Antibody based Therapy:**

- CD3 antibody therapy
- CD20 antibody therapy

**Others:**

- DNA Vaccine using GAD and Microsphere
- Cyclosporin
- Vitamin D

Some of the promising approaches are:

## Alum Formulated GAD based Immunotherapy

Phase II b trials in T1D patients with alum formulated GAD showed significant preservation of beta cell function 30 months after the first 20 μg dose administrations. It also induced antigen specific T cell population, cytokines involved in regulation of immune system and a long-lasting B cell memory, suggesting that modulation of general immune responses to GAD can be helpful in preserving residual β-cells.[6]

Alum formulated GAD is the only antigen-based vaccine candidate which has been shown to be effective in both T1D and Latent Autoimmune Diabetes in Adults or LADA. In LADA, five year follow-up results have now been published in Diabetologia, (2009) which showed excellent beta cell preservation. In vaccine doses of 20 ug and 100 ug, vaccinated patients only 14% required insulin at five years compared to 70 to 80% needed insulin in placebo, 4 ug and 500 ug doses.

Several ongoing studies (listed below) will make the vaccine useful in established T1D patients: (a) Large-scale phase III clinical trials are being conducted in Europe and the United States to confirm these findings. When completed and if approved, the vaccine is likely to reach the market in 2011, (b) Approval has also been obtained from the Swedish Drug controller authorities for using the vaccine in prevention of T1D in children at risk for developing the disease. (c) Norwegian Drug controller Agency has given approval for the GAD vaccine to investigate the disease process before T1D onset and if treatment with GAD vaccine stops the process. A unique feature of this study is that tissue samples from pancreas are allowed to be taken. Analysis of these tissue samples can provide direct insight into how GAD vaccine works to reduce beta cell destruction. This study will include 90 adults at high risk for developing T1D and additional 60 recent onset T1D patients. (d) Most interesting aspect of the new study (approved by FDA) is to treat established T1D in children with vaccine for beta cell regeneration either alone or in combination with beta cell regenerative agents like: Lansprazole and sitagliptin.

This study is funded and carried out by NIH in Bethesda, USA. If shown to be successful, this will allow GAD vaccine to be given to all recent and old-onset T1D for beta cell regeneration. This vaccine is produced by the Swedish company, Diamyd.

## DNA- based Antigen specific Immunotherapy using Proinsulin

The candidate proinsulin vaccine is called BHT-3021 which is a DNA based antigen specific immunotherapy currently in Phase I/II clinical trial in patients with T1D. BHT-3021 is a plasmid encoding proinsulin, designed to target specific pathogenic immune cells responsible for the autoimmune response in type 1 diabetic patients. The compound has shown efficacy in NOD mice, a model of type 1 diabetes. In the current phase I/II trial, patients receiving BHT-3021 demonstrated preservation of C-peptide and an acceptable safety profile. BHT-3021 is designed to induce antigen specific tolerance by selectively turning off the errant autoimmune response attacking the pancreas. This highly specific immuno-modulation action could result in the preservation of pancreatic function and improved long term health in type 1 diabetic patients.

BHT-3021 was the subject of a recent presentation by Dr. Peter Gottlieb of the Barbara Davis Center at the American Diabetes Association's 69^th^ Scientific Sessions in New Orleans. The abstract, entitled “Interim Results of a Phase I/II Clinical Trial of a DNA Plasmid Vaccine (BHT-3021) for type 1 diabetes,” presented interim results from a randomized, blinded, placebo- controlled, dose escalation trial in T1D patients. Patients were randomized 2:1 to receive a weekly intra-muscular injection of BHT-3021 (0.3 mg, 1 mg, 3 mg or 6 mg) or placebo for 12 weeks. Data presented included pancreatic function as measured by C-peptide and safety data from a total of 42 C-peptide positive patients. Data from the 1 mg dose cohort was available out to 12 months, and data from the 0.3 mg, 3 mg, and 6 mg dose groups were available out to six months. Patients in all 4 dose cohorts exhibited a preservation of C-peptide compared to placebo. Adverse event (AE) and clinical laboratory data indicate that BHT-3021 is safe and well- tolerated. No treatment-related serious adverse events have been reported. Most AEs were mild or moderate in severity. This product is developed by a US based company called Bayhill. Therapeutics and Genentech have bought the license to develop and market throughout the world.

## Antibody-based immunotherapy for T1D

### CD3 monoclonal antibody therapy

Modified anti-human CD3 monoclonal antibodies was thought to be the next alternative to previously used OKT3 antibody for the prevention of T1D in NOD mouse. Mouse monoclonal antibody caused serious AE which were infusion releted. Fc-mutated (Fc-nonbinding) monoclonal human CD3 antibodies were engineered and these were found to be less mitogenic, but were equally tolerogenic compared to functional Fc CD3 antibodies.[7,8] Two humanized CD3 antibodies are in different stages of clinical trials. They are the ChAglyCD3 antibody developed by Tolerx, USA (www.tolerx.com), with a single mutation (Asn→Ala) at residue 297 in the Fc region that prevents glycosylation, derived from rat YTH 12.5 antibody[9] and the hOKT3Ala-Ala antibody developed by Macrogenics, USA (www.macrogenics.com), with two mutations at residues 234 (Lue→Ala) and 235 (Lue→Ala) in the Fc region.[10]

### CD20 monoclonal antibody therapy

Until recently B cells were thought to play an important role in priming T cells.[11] However, a recent study showed for the first time that B cells promote the survival of CD8+ T cells in the islets and there by promote the disease.[12] CD20 is a cell surface marker expressed on all mature B cells. Rituximab (Roche/Genentech), a humanized anti CD20 monoclonal antibody (CD20 mAb) has been shown to successfully deplete human B cells from peripheral circulation via mechanisms involving Fc and complement mediated cytotoxicity and probably via proapoptotic signals.[13,14] Given the important role of B cells in maintaining the T1D, depleting B cells is a very interesting therapeutic option. TrailNet is currently testing the efficacy of rituximab (CD20 mAb) in a new-onset trail involving four week course treatment with the antibody.[15] This therapy has been developed by Genentech, now owned by Roche.

There is real hope for T1D patients both in early diagnosis and prediction as well as beta cell preservation and beta cell regeneration.
